# Chicago Medical Society

**Published:** 1875-08

**Authors:** 


### CHICAGO MEDICAL SOCIETY.

*Transactions at Regular Meeting, June 21st, 1875.*

The President, Dr. William E. Clark, in the chair.

In the absence of the regular reporter, Dr. Charles Warrington Earle was appointed for the evening.

The minutes of the preceding meeting having been read, and certain preliminary business transacted, the following paper by Dr. Norman Bridge was read:

### *Do Mental Emotions in a Pregnant Woman produce Deformity in her Child?*

The medical profession have given little attention, in the way of direct study, to this subject. A few essays have been written, and a goodly number of cases bearing upon the question recorded, but most students when cornered for a positive opinion have evaded the point, and given a statement that concedes the possibility of both or either side being correct.

But there is a widespread popular belief that all kinds of deformities may come to unborn children, all kinds of marks and disfigurements, from such causes as a fright, an erratic thought, or any sudden, unusual or marked mental impression made on the mind of the gestating mother.

Reports of thousands of cases of such sequence pass from mouth to mouth among the people, and every case is so convincing in its minute and accurate statements that few people presume to doubt the truth of the laws they seem to be governed by.

Many good and conscientious physicians join in this belief; they believe that they found their opinions on fact. This adds to the strength of the popular opinion vastly, for it always happens that a few of the choicest minds in a community will always follow the opinions of their cultivated doctors. So general is the popular belief in the possibility of damaging the child by wrong mental emotions on the part of the mother during its intra-uterine life, that there is a quite general carefulness on the part of pregnant women against excitement and mental disturbances of all kinds and in favor of a peaceful, tranquil and happy passage through this period of woman's experience. In deference to this opinion, too, and in addition to the natural instincts, husbands and friends are more considerate and careful of gestating women.

All this is most proper and commendable. But as a consequence of the belief, it often happens that women who know they have experienced profound and often painful emotions during the early months of pregnancy, are made more than miserable through the remaining weeks for fear their children *will* be deformed or marked. The expressions of this suffering are the frequent observation of every physician. The suffering is often severe and distressing; it amounts to the worst possible mental agony.

I feel certain that in the aggregate the worry and suffering on this account, far exceeds that from any fear of the dangers and uncertainties of parturition. Whatever the preventive effect of this popular belief may be, the consternation on the part of gestating women for fear injury *has been done*, is a hardship so common and so appalling that I think it ought to be a subject of study with the profession whether there is any good ground for the notion.

If it is untrue, it comes to be almost a criminal cruelty to aid in its propagation. If it is untrue, it does no good; while the injury it works is so considerable that no word should be spared against it.

If it is true, of course it should not be denied; but then in that case it is a proper question whether the evil it does is not so much greater than the possible good, that anything that will aid the spread of it is unjustifiable. We study to avoid having patients with mortal diseases for months before death become fully aware of their incurability. Human peace and happiness are so precious a boon that we evade the queries of these unfortunates and laugh at their misgivings, and divert them with conversation and attentions and gifts. Why not then, if the belief in question be true,—why not fill the heads of people with so much beside, that they cannot think of it; why not switch them off from their musing on the subject and laugh at them for their devotion to a questionable notion that can only stab them?

But the personal belief of the writer hereof is against the truth of the conviction; and this is so strong and positive that it makes the popular view assume an aspect not creditable for scientific people to hold, and betrays him, in characterizing it, into the use of language that is sometimes neither becoming in him who uses it nor respectful to the people it hits, who are too often his professional seniors.

Dr. G. J. Fisher wrote some years ago an article opposed to the idea that mental emotions in gestating women cause deformities in their children. He declared that most women during gestation have intense emotions and may see sights of awful and horrid character, yet deformed children are few ; furthermore, in many cases, that is, where there was fright and no deformity, the mothers have expected their offspring to be monstrosities. I think any candid observer would say he has known of twenty women who had seen sights or had emotions and expected their babies to be deformed at birth, to every one who had borne a deformed child. "Like causes produce like results, yet the most widely dissimilar causes are assigned for the same deformity." All sorts and grades of emotions are invoked to explain a given deformity, and the emotions and their causes frequently have nothing about them to suggest the deformity. When a woman sees a hog with its throat cut and bears a child with a red mark on its throat, the theory of cause and effect looks plausible ; but when the same kind of a fright is offered as a cause of an idiotic child, our concurrence may fairly be reserved. I have no doubt that in a majority of cases "the most widely dissimilar causes are assigned for the same deformity."

In a large proportiou of the malformations no explanation is offered by the parents. Is it possible that so vivid emotions as can cause such deformities as we are told about and see, can be so easily forgotten by parents who are human ? Mankind all love mystery and wonderful occurrences !

There is no doubt that in very many cases, probably four in every five, the explanation of a deformity by a mental emotion is an afterthought. *After* the birth of the child reveals a deformity, the woman asks herself if she had not seen a sight, been frightened, or had some other emotion, and of course it is easy to recall such a circumstance. I believe the cases where an emotion has occurred and a deformity is expected and it really occurs, and of a character that could possibly be suggested by the nature of the emotion, are almost unheard of, and further, that in not one in a thousand of the reported (popularly reported) cases that are all remembered and circulated, are all these points inquired into. But the most awkward inconsistency of the claim that such things are possible is the fact that the date of the deformity of a part due to an emotion that occurred at a known period, is frequently *after* the part in the course of fœtal development must have been formed. A woman sees a man who is minus one leg, she is in a stage of pregnancy many weeks past the time when the lower extremities of the fœtus are known to be fully formed; the baby is born maimed in one of its legs.

If the sight caused the deformity, there must have been a veritable amputation. Well, Dr. Seguin says, I believe, that mental emotions may cause amputation of parts of the fœtus in utero. But has he or anybody else ever discovered the amputated limb? If fingers, toes, ears, arms and legs are amputated in the later months of utero gestation, what becomes of them?

Seguin says also that deep gashes (wounds) may be made in the fœtus by mental emotions in the mother. This might be supposable could it be demonstrated that nerves connect the spinal cord of the mother directly with that of the fœtus. Dr. Tuke mentions some cases where mental emotions in sensitive people have led to great textural changes in the parts of the individual on which the mind was directed. But no such nervous connection has ever been made out, and it is denied on authority.

Dr. Fisher declares that malformations identical in kind and degree recur often in the human subject and admit of classification, and that they have their exact morphological counterparts in the lower animals, as well as some that are analogous in the vegetable kingdom.

Note the frequency with which cleft palate and harelip occur, and spina bifida. Would any one claim that these defects are due to mental emotion in the mother during utero gestation?

[The Doctor here quoted a large number of cases from the writings and observations of such men as Tuke, J. L. Smith, Hammond, Dalton, Carpenter, Brown-Sequard, and others, resuming his paper with the following apology and enumeration of cases in his own practice.]

I may be pardoned in referring to a few cases that have come under my eye and to my knowledge in the slight experience I have had in this branch of medicine.

Mrs. G. M. D. after a peaceful and tranquil pregnancy gave birth to a child with a spina bifida. Both parents were healthy, and there had been no unusual emotions. The woman was the daughter of a physician. One child before and one since have been born and both perfectly healthy.

Mrs. ——, a healthy young woman, after a pregnancy marked by no emotions but those of perfect happiness, gave birth to a spina-bifidous child. She has since borne a healthy and normal child.

Mrs. ——, on the very day of the birth of the last mentioned deformed child, brought forth a child absolutely without a blemish. In the early months of the gestation she had seen her own husband kill a man, and had from that moment been unspeakably miserable in the firm belief that her child would be deformed.

On the 2nd of May, *ultimo*, Mrs. K. was delivered of her first child. She is a sensible woman but a sensitive one, and lived through most of her pregnancy in the active fear that her child would be a monster, because she had seen a dead-born, half-grown fœtus in the earlier months of her gestation. Her child, so far from being a monster, is the embodiment of beauty and symmetry.

These cases are gathered without effort. Nearly every member of the profession, if he will recall cases that have fallen under his observation, could muster a much larger array than I have—enough, at least, to convince him that the theory in question is a chimera.

But it is urged that, as the first offspring of the mother in a measure determines the character of her after issue, it somehow explains or establishes the theory we are discussing. It is known that children of a second marriage often resemble the first husband, provided the first marriage has been fruitful. A negress bearing her first child to a white man, bears light colored children afterward to a black man.

We are told a Zebra once impregnated a mare who afterward bore striped colts.

Stock raisers discard a mare or cow for the raising of blooded stock if she has borne the first offspring to an inferior animal.

Now it is claimed that these facts prove that the most wonderful influences can be carried through the mother's blood to the child in utero; that such is the manner of the effect made on the later offspring referred to.

But it is not proved, and it is highly improbable, that the mother transmits the qualities of either her father or her former husband to her children through the medium of her blood. It is far from rational that the resemblance of the product or offspring to the parent—characteristic of all animals and plants—is transmitted directly through so accidental and mutable a thing as the floating medium called blood. What of the animals that have no blood?

The blood is accidental; *cells* of tissue are all potent and constant, and *the*, and the only characteristic, cells reproduce their kind; nothing else on earth does.

If a woman bears children resembling a former husband, it simply *must be* because her tissues, not her blood, probably the ova in her ovaries, possibly her uterus, have had their characteristics changed by her former pregnancy.

The fact is, the only rational view we can take of the subject in the light of all our teaching on physiology and pathology is, that when a deformity or monstrosity occurs, it is due to either an abnormal state of the generative matter of one or both parents at the beginning, or to some obstruction to the normal development of the fœtus. Development may be retarded—may be excessive, or perverted. A thousand different forms of disturbance might occur and each be capable of interfering with perfect development. There might be too much or too little blood supplied the fœtus; the quality of this fluid might be abnormal in countless ways. Accidents may occur, blows and falls. There may be frequent attempts at miscarriage, or the mother may be syphilitic or may have some acute disease. The maternal organism may be abnormal; so may the membranes, or the cord—indeed, it would be useless to attempt to give all the influences capable of arresting or disturbing the development of the ovum.

Mental emotion is such a cause; that is, it is a cause of disturbed nutrition, able to work a failure of a perfect development of the fœtus. It may act on the fœtus as it does on the stomach of the individual; if it disturbs the character of the blood, all parts of the body of the mother and of the fœtus alike would suffer in some measure.

But mental emotion does not act through the blood to disturb, in the most wonderful manner, a particular part of the body of the fœtus, the part varying according to the particular mental impression. To endow the blood with such wierd intelligence as this would require, is too great a load for our credulity. There is no philosophy in the theory that it so acts; all the truths of anatomy, physiology and pathology are directly against it. It is in nature more nonsensical than the theory that the decillionth of a grain daily of sulphur will cure hip-joint disease. It has nothing in its favor except the fact that in an occasional case a deformity occurs as, judging from an emotion really experienced, the mother expected it would. But the cases of this kind are no more frequent than, by the rule of possibilities, the mathematicians would tell us they must occur in the course of events, as the inevitable accident, the coincidence. By a similar system of figuring, they would aver that, in a large and busy city like Chicago, with all the dangers incident to travel and business, and with the native human carelessness in the face of familiar dangers, out of each quarter million of people a given number would be sure, each year, to have their legs broken, and they would undertake to tell us whether most of the fractures would be on the right or left side of the body.

But if the popular belief is true, it is so near a falsehood that, in the interest of pure humanity, the profession ought to turn their faces against it.

The profession ought everywhere, and on all occasions when the subject is broached, to deny the probability of the notion.

From beginning to end the belief partakes of the nature of stories that are published in newspapers, and pass from mouth to mouth, growing as they go; which have no philosophy or reason for their foundation. They start out of the necessity on the part of little thinking people of hatching up some single and tangible cause to explain every peculiar occurrence, regardless of whether such be *capable* of explanation, out of the towering necessity of having a scape-goat for everything; they are remembered and retailed to everybody, and enlarged, through the mania, on the part of the same little thinking people, for always having some wonderful thing to tell or to hear.

The profession ought to be no longer chargeable with propagating such a delusion.

In the discussion which followed the paper, Dr. Paoli, misunderstanding the ground taken by the author, referred to many of the foolish theories believed by the people, and placed the opinion received by many, that mental emotions in the pregnant mother produce marks and deformities in her offspring in the same category.

Maimed men, many of whom we knew since the war, marry and beget healthy and perfectly formed children. He believed that bad air, bad hygenic regulations, bad nutrition, etc., were the causes which produced the deformities mentioned in the paper.

Dr. Hutchinson—than whom there is no one more able—recalled the biblical knowledge of the Society. He spoke of the efforts made centuries ago, to produce certain peculiarities in the offspring of the lower animals. He could not give a reason why children were born deformed and marked, but thought it was bad nutrition. The same speaker related cases in his own practice, where the birth of deformed children were followed by the perfectly formed and healthy.

Dr. J. S. Knox remarked that it was an established fact, he believed, that no nervous connection existed between the mother and child. Any deformity existing must be the result of bad nutrition.

Dr. Lee related the history of several cases of marked and deformed children, giving the reasons assigned by the parents.

Dr. Engert believed that deformities were inherited the same as family resemblances.

Dr. Stevenson believed that certain members of the profession had strengthened the opinion in the minds of the people, and referred particularly to a novel published some years ago, the author of which was not only a physician but a gentleman of rare literary acquirements. The book represented * * * * * and had a large sale, and undoubtedly influenced the people to a considerable degree, coming, as it did, from a physician. It seemed to her much easier to explain marks and deformities than to account for hereditary transmissions.

The President said that it was not wonderful that the people believed deformities, etc., were produced by mental emotions; indeed nothing was too outrageous as not to find some to readily accept it. Men carried dried potatoes in their pockets for years, and believed them a sure preventive against rheumatism. Horse-shoes were displayed above the doors, in dwellings, where in every thing else the occupants appeared rational and sensible. The medical delusion, Homœopathy, was advocated and championed by many who, on all other subjects, seemed to be possessors of a well balanced and cultivated intellect.

At a late hour the Society adjourned, to meet in three weeks at the residence of the President.

